# Protection of Si Nanowires against A*β* Toxicity by the Inhibition of A*β* Aggregation

**DOI:** 10.3390/molecules29091980

**Published:** 2024-04-25

**Authors:** Xuechun Zhao, Chenye Mou, Jiayi Xu, Wei Cui, Yijing Shi, Yangzhe Wang, Tian Luo, Wei Guo, Jichun Ye, Wanghua Chen

**Affiliations:** 1 School of Physical Science and Technology, Ningbo University, Ningbo 315211, China; 2111077058@nbu.edu.cn (X.Z.); shiyijinghaha@163.com (Y.S.); 13306828588@163.com (Y.W.); 2 Zhejiang Provincial Key Laboratory of Pathophysiology, Health Science Center, Ningbo University, Ningbo 315211, China; mcynb123@163.com (C.M.); 18857509352@163.com (J.X.); 3 Ningbo Institute of Materials Technology and Engineering, Chinese Academy of Sciences, Ningbo 315201, China; luotian@nimte.ac.cn (T.L.); guowei@nimte.ac.cn (W.G.); jichun.ye@nimte.ac.cn (J.Y.)

**Keywords:** Alzheimer’s disease, amyloid *β*, silicon nanowires, PC12 cells, toxicity

## Abstract

Alzheimer’s disease (AD) is a progressive neurodegenerative disease characterized by the accumulation of amyloid beta (A*β*) plaques in the brain. A*β*_1–42_ is the main component of A*β* plaque, which is toxic to neuronal cells. Si nanowires (Si NWs) have the advantages of small particle size, high specific surface area, and good biocompatibility, and have potential application prospects in suppressing A*β* aggregation. In this study, we employed the vapor–liquid–solid (VLS) growth mechanism to grow Si NWs using Au nanoparticles as catalysts in a plasma-enhanced chemical vapor deposition (PECVD) system. Subsequently, these Si NWs were transferred to a phosphoric acid buffer solution (PBS). We found that Si NWs significantly reduced cell death in PC12 cells (rat adrenal pheochromocytoma cells) induced by A*β*_1–42_ oligomers via double staining with 3-(4,5-dimethylthiazol-2-yl)-2,5-diphenyltetrazolium bromide (MTT) and fluorescein diacetate/propyl iodide (FDA/PI). Most importantly, pre-incubated Si NWs largely prevented A*β*_1–42_ oligomer-induced PC12 cell death, suggesting that Si NWs exerts an anti-A*β* neuroprotective effect by inhibiting A*β* aggregation. The analysis of Fourier Transform Infrared (FTIR) results demonstrates that Si NWs reduce the toxicity of fibrils and oligomers by intervening in the formation of *β*-sheet structures, thereby protecting the viability of nerve cells. Our findings suggest that Si NWs may be a potential therapeutic agent for AD by protecting neuronal cells from the toxicity of A*β*_1–42_.

## 1. Introduction

Alzheimer’s disease (AD) is a progressive neurodegenerative disorder characterized by the irreversible decline of cognitive function. Notably, AD stands as the most prevalent form of dementia, afflicting millions of patients globally. The main features of AD involve the pathological accumulation of misfolded protein aggregates, predominantly amyloid-*β* (A*β*) plaques and tau neurofibrillary tangles, within the central nervous system. These aberrant protein assemblies trigger a cascade of neurotoxic events, culminating in widespread neuronal death and the progressive disorganization of neuronal networks [1]. Peri-neuronal deposition of A*β* aggregates disrupts inter-neuronal communication, compromises network function, and leads to cognitive and memory deficits. Notably, these aggregates further trigger neurodegeneration by inducing neuroinflammatory responses [2] and amplifying oxidative stress [3], both promoting neuronal death and network disorganization. Therefore, there is a focus on deciphering and abrogating A*β* aggregation for AD therapy. Numerous investigations are actively exploring therapeutic strategies, including the inhibition of aggregate formation and the exploitation of their potential as therapeutic targets.

Enzymatic cleavage of the amyloid precursor protein (APP) generates A*β* [4]. Differential cleavage patterns yield various A*β* proteins, ranging from 39 to 42 amino acids in length, with A*β*_1–42_ displaying the highest aggregation. A*β* aggregation, a complex multi-step process, entails nucleation and elongation phases [5]. During nucleation, A*β* assembles into small, highly toxic oligomers, which represent key players in neuronal death and network dysfunction [6]. Elongation involves the coalescence of these oligomers into larger amyloid plaques that hinder interneuronal communication. Several factors drive A*β* aggregation, for example, hydrophobic interactions within the fibril core [7,8], electrostatic interactions that stabilize *β*-sheets [9], *β*-folding characteristic of amyloid fibrils [8,10], dysregulated protein degradation and chaperone-mediated misfolding in protein quality control [11], as well as oxidative stress-induced misfolding through free radicals [12].

Existing efforts to mitigate A*β* aggregation primarily focus on small molecule therapy (e.g., A*β* aggregation inhibitors [13,14] and immunotherapy [15]). Recently, nanomaterials have emerged as promising candidates for novel anti-A*β* agents due to their advantageous properties, including small size and high surface area. Numerous nanomaterials have been explored for their A*β*-modulating potential, including magnetic nanoparticles [16], carbon nanotubes [17,18], Au nanoparticles [19], and graphene-based 2D materials [20]. For example, the magnetoelectric material BCFO nanoparticles successfully dissociated A*β* aggregates and demonstrated the mitigation effect of BCFO nanoparticles on A*β*-related toxicity [16]. Au nanorods (Au NRs) can also dissociate A*β*_1–42_ by triggering ultra-high local surface plasmon resonance (LSPR) heating, destroying mature *β*-amyloid fibril at full length within minutes [19]. In the study of the interaction between single-walled carbon nanotubes (SWNTs) and amyloid protein, it was found that the strong hydrophobic and aromatic accumulation interaction between CNTs and A*β* peptide significantly inhibited the formation of *β*-sheet structure, and then inhibited the process of A*β* fibrosis [21]. It was also found that SWCNTs promoted nucleation of A*β* peptides and guided the formation of a new class of non-amyloid fibril with A*β* peptides [22]. For pre-formed A*β* fibril, SWCNTs can partially destroy it and form A*β*-SWCNTs complexes, and reduce the *β*-sheet structure [23]. However, several challenges remain. Their tendency to aggregate and form clusters in vivo can hinder their dispersion and efficacy in A*β* clearance. Additionally, despite their noted benefits, certain nanomaterials exhibit potential toxicity. For instance, Au nanoparticles are recognized for their potent antioxidant and anti-inflammatory capabilities [24], yet they have also been found to induce apoptosis in human nerve cells [25]. Similarly, unmodified carbon nanomaterials can trigger cytotoxicity and inflammatory responses. Specifically, carbon nanotubes, even at low concentrations of 0.0008 mg/mL [21], can cause the death of most cells. Furthermore, larger sizes of carbon nanotubes have been reported to induce deformation of the cell wall in living cells, resulting in cell death. These limitations severely restrict the in vivo implementation.

Overcoming the inherent limitations of existing anti-A*β* agents is crucial for advancing AD therapeutics. Si, established in the biomedical and bioengineering fields for its biocompatibility [26,27,28], offers a promising alternative. Particle size significantly influences nanomaterial aggregation. Smaller particles, with larger surface areas and stronger interparticle interactions, tend to aggregate more readily. Si nanowires (NWs) can be synthesized by chemical vapor deposition (CVD) via vapor–liquid–solid (VLS) mechanism using Au as catalysts [29,30,31,32]. As far as the bio domains are considered, Si NWs are widely studied. For example, the design of conductive Si NW for cardiac-like organs significantly enhanced the therapeutic effect of human pluripotent stem cell-derived cardiomyocytes (hPSC-CMs) in treating infarcted hearts [33]. In terms of drug delivery, Peng et al. utilized a freestanding Si NW with a diameter of about 100 nm and a length of about 500 nm to load the anticancer drug DOX [34]. As typical one-dimensional nanostructures, NWs can directly penetrate individual cells, effectively delivering payloads within cells without any external force, thereby bypassing the limitations of traditional cellular absorption of 0D particles [35,36,37].

In the case of the application of A*β*, the elongated directional shape weakens NW interactions, thereby reducing their aggregation. In order to explore the interaction between Si NWs and A*β*, as well as the protective effect of Si NWs on neuronal cells, in this study, we investigated the impact of Si NWs on A*β* self-assembly and fibril formation, employing characterization techniques such as scanning electron microscopy (SEM), atomic force microscopy (AFM), attenuated total reflectance Fourier Transform Infrared (ATR-FTIR), and transmission electron microscopy (TEM). We further explored the ability of Si NWs to mitigate the toxicity of A*β*_1–42_ oligomers in vitro.

## 2. Results and Discussion

### 2.1. Characterization of Si NWs

**Figure 1:**
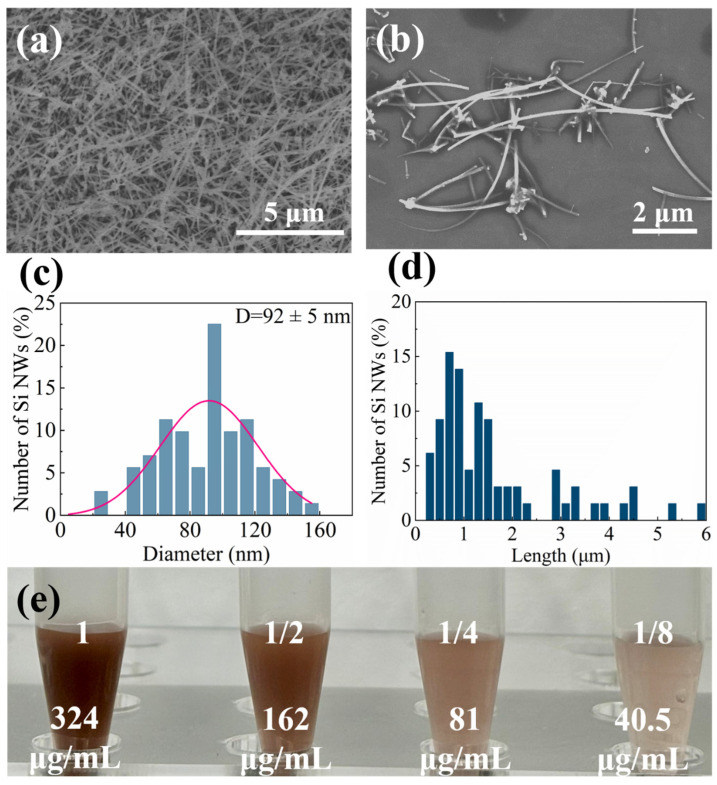
SEM reveals the morphology and dimensions of the Si NWs. (**a**) Original array of Si NWs grown on a silicon substrate. (**b**) Si NWs transferred onto a silicon substrate. Both images have a scale bar of 2 μm. Additional characterization of the Si NWs is shown in (**c**) width distribution and (**d**) length distribution. (**e**) Illustrations of Si NWs at different final concentrations in PBS: “1 concentration” (324 μg/mL), “1/2 concentration” (162 μg/mL), “1/4 concentration” (81 μg/mL), and “1/8 concentration” (40.5 μg/mL).

Figure 1a,b present the morphology and dimensions of Si NWs used in this study. Figure 1a displays the as-grown Si NWs on a Si wafer, while Figure 1b showcases the transferred Si NWs on a Si substrate. The average width of the Si NWs is 92 ± 5 nm, and their lengths range from hundreds of nanometers to a few micrometers, as shown in Figure 1c,d. Figure 1e features illustrations of Si NWs at different final concentrations employed in the experiment: 324 μg/mL (“1 concentration”), 162 μg/mL (“1/2 concentration”), 81 μg/mL (“1/4 concentration”), and 40.5 μg/mL (“1/8 concentration”). It is observed that there is no clustering and the liquid’s color progressively lightened with decreasing concentration.

### 2.2. Inhibition and Remodeling of Aβ_1–42_ Fibrils by Si NWs

**Figure 2:**
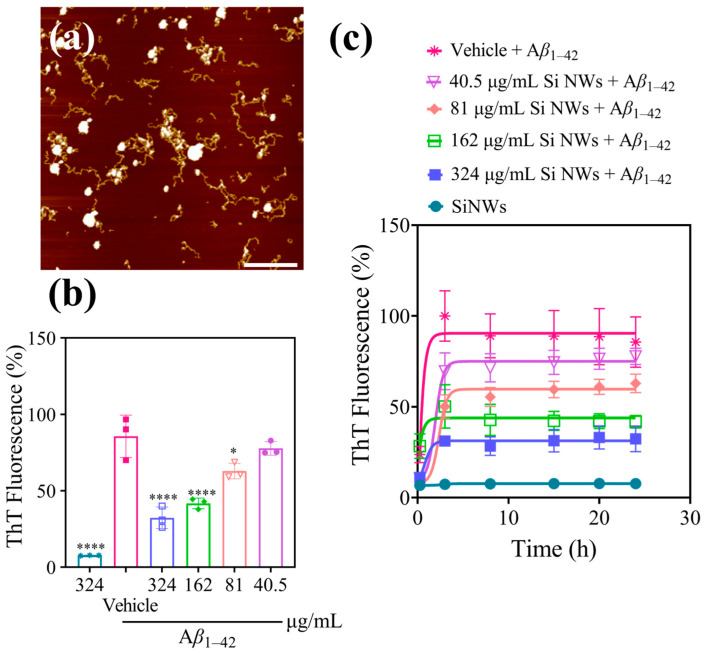
(**a**) AFM image of A*β*_1–42_ monomers (10 μM) incubated with PBS (control) for 24 h under fibrillating conditions. Addition of Si NWs inhibited A*β*_1–42_ fibril formation. (**b**) Concentration-dependent inhibition of A*β*_1–42_ aggregation by Si NWs was evaluated. A*β*_1–42_ monomers (10 μM) incubated with PBS (control) or varying Si NW concentrations (40.5–324 μg/mL) for 24 h under fibrillating conditions were assessed using ThT fluorescence. (**c**) The time-dependent effect of Si NWs on A*β*_1–42_ fibrillization was investigated. A*β*_1–42_ monomers (10 μM) incubated with PBS (control) or varying Si NW concentrations (40.5–324 μg/mL) for different incubation times under fibrillating conditions were assessed using ThT fluorescence. Data are presented as mean ± standard error of mean (*n* = 3). Statistical significance was assessed using one-way ANOVA followed by Tukey’s post hoc test. Asterisks denote significant differences compared to the Vehicle group: * *p* < 0.05, **** *p* < 0.0001.

It is reported that thioflavin-T (ThT) selectively binds to amyloid fibrils, enhancing their fluorescence intensity [38]. Si NWs exhibits a dose-dependent inhibitory effect on A*β*_1–42_ fibrillization in vitro (Figure 2a). The ThT fluorescence intensities of different concentrations of Si NWs (40.5, 81, 162, and 324 μg/mL) co-incubated with A*β*_1–42_ monomers were 78%, 63%, 42%, and 32% of the fluorescence intensity of the A*β*_1–42_ monomer group, respectively (Figure 2b). This observation was further confirmed by monitoring the time course of A*β*_1–42_ aggregation in the presence of 10 μM A*β*_1–42_ monomer (Figure 2c). The typical ThT fluorescence intensity curve, showcasing distinct hysteresis, polymerization, and saturation stages, reached a plateau within ~8 h, consistent with the reported A*β*_1–42_ fibrillization kinetics [39]. Notably, compared to the control, Si NWs at all tested concentrations significantly suppressed the ThT fluorescence signal throughout the aggregation process, demonstrating their capacity to inhibit A*β*_1–42_ fibrillization. To verify that the observed ThT fluorescence reduction solely arose from A*β*_1–42_ fibrillization inhibition without Si NW-ThT binding, a control experiment was performed. Only Si NWs (324 μg/mL) and ThT dye were added to the system. The resulting minimal fluorescence (7.7% of A*β*_1–42_ monomer control) confirms negligible direct interaction between Si NWs and ThT, validating the observed effects in co-incubation experiments as accurate reflections of A*β*_1–42_ fibrillization inhibition by Si NWs. The results validate that the reduced ThT fluorescence in co-incubation experiments accurately reflects A*β*_1–42_ fibrillization inhibition by Si NWs. While the optimal Si NW concentration for suppressing ThT fluorescence in this study (324 μg/mL) was higher than that reported for chiral L/R-SiO_2_ nanoribbons (200 μg/mL) [40], the 162 μg/mL group exhibited a similar inhibitory effect. These findings suggest the potential efficacy of Si NWs as an alternative for A*β*_1–42_ aggregation modulation.

**Figure 3:**
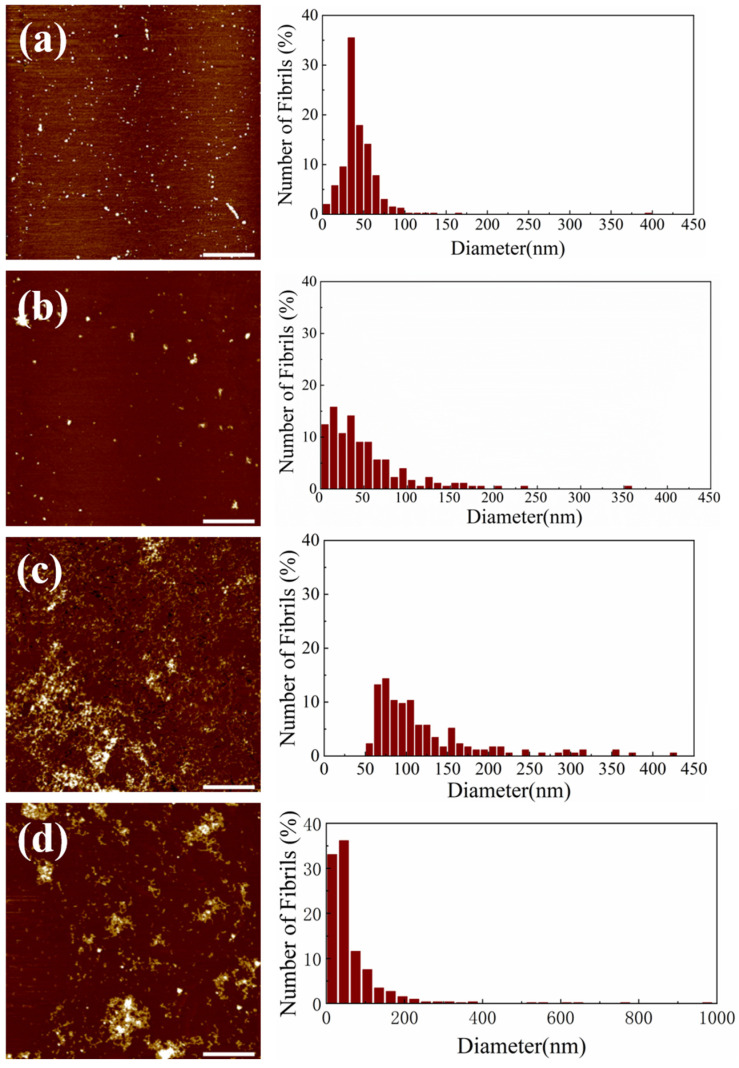
AFM images of A*β*_1–42_ monomer (10 μM) incubated with PBS (vehicle) and Si NWs (40.5–324 μg/mL) for 24 h under fibrotic conditions: (**a**) 324 μg/mL; (**b**) 162 μg/mL; (**c**) 81 μg/mL; (**d**) 40.5 μg/mL. The corresponding particle size distribution histogram is shown on the right. The scale bar is 1 μm.

AFM imaging reveals distinct effects of Si NWs on A*β*_1–42_ fibrillization at varying concentrations under fibrillizing conditions (Figure 3a–d). These figures show AFM images and corresponding particle size distributions obtained by adding different Si NW concentrations (324, 162, 81, and 40.5 μg/mL, designated as “1 concentration,” “1/2 concentration,” “1/4 concentration,” and “1/8 concentration,” respectively) to A*β*_1–42_ monomers during fibrillization. Compared to Figure 2a, the fibril morphology exhibits varying degrees of disruption depending on the Si NW concentration. At the highest concentration (324 μg/mL), fibrils are virtually absent, replaced by small particles with an average diameter of 37 ± 1 nm, as confirmed by the accompanying histogram. Upon decreasing the Si NW concentration to 162 μg/mL (“1/2 concentration”), some small *β*-amyloid patches with diameters of 100–200 nm emerge alongside the smaller particles. Interestingly, at the even lower concentrations of 81 and 40.5 μg/mL (“1/4” and “1/8 concentration”), fibril formation progressively increases, although complete inhibition is not achieved. These observations demonstrate a dose-dependent intervention of Si NWs on A*β*_1–42_ fibrillization, suggesting their potential as concentration-dependent modulators of amyloid aggregation.

Figure 3c–d showcases a progressive shift in A*β*_1–42_ aggregation patterns with decreasing Si NW concentrations. Compared to Figure 3a,b, the dominant morphology transforms from small particles to large (50–200 nm) plaques with interspersed short fibers. At the lowest concentration (40.5 μg/mL, Figure 3d), these plaques reach their maximum size (near 1 μm) and are densely surrounded by short fiber networks. Interestingly, Figure 3d presents a subtle deviation from this trend. While plaques remain large (slightly wider than in Figure 3c), they appear more dispersed. This suggests that at this minimal Si NW concentration, protein aggregation proceeds to some extent, resulting in larger, but sparser entities. This is further supported by the measured volume of these hybrid patches in Figure 3d, which is the highest among all groups, potentially reflecting the combined volume of NWs and aggregated proteins. These observations collectively demonstrate the concentration-dependent ability of Si NWs to modulate A*β*_1–42_ fibrillization. Their presence disrupts complete fibril formation, favoring the assembly of larger, but less organized, plaques at lower concentrations. This potential for modulating amyloid aggregation patterns warrants further investigation. Quantitative analysis of Figure 3d reveals the largest hybrid patch volume alongside less dense surrounding fibers. This suggests that while Si NWs at the lowest concentration (40.5 μg/mL) partially hinder fibrillization, some oligomer and fiber assembly still occurs independently. Overall, these observations demonstrate the concentration-dependent efficacy of Si NWs in modulating A*β*_1–42_ self-assembly. Their presence disrupts complete fibril formation, favoring smaller, dispersed entities as the concentration decreases.

### 2.3. Si NWs Reduce the Toxicity of Preformed Oligomers of Aβ_1–42_ In Vitro

**Figure 4:**
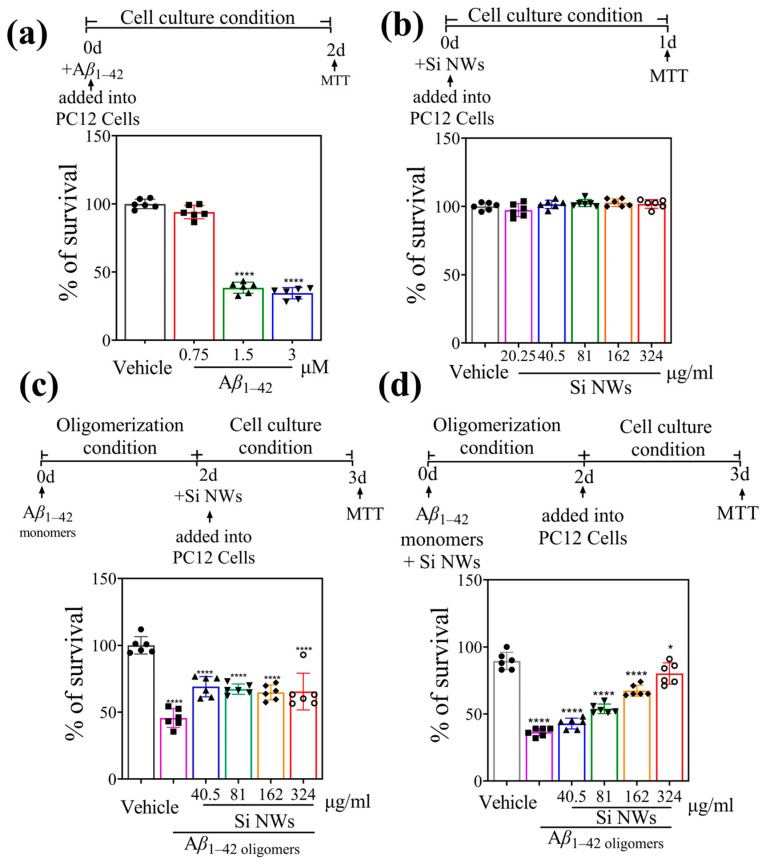
(**a**) A*β*_1–42_ dose-dependently produce cytotoxicity in PC12 cells. PC12 cells were treated with increasing concentrations of A*β*_1–42_ oligomers (0.75–3 μM) for 48 h, and cell viability was assessed using the MTT assay. PBS served as the control treatment. (**b**) Si NW does not produce cytotoxicity in PC12 cells. The MTT assay was also performed on PC12 cells treated with varying Si NW concentrations (20.25–324 μg/mL) for 24 h to establish potential cytotoxicity of the nanowires themselves. (**c**) Pre-incubation with Si NWs produces anti-A*β* neuroprotection in PC12 cells. PC12 cells were pre-incubated with Si NWs (40.5–324 μg/mL) for 2 h, followed by A*β*_1–42_ oligomer (1.5 μM) addition and incubation for 24 h before MTT assay. (**d**) Si NW co-incubation prevents A*β*-induced toxicity in PC12 cells. A*β*_1–42_ oligomers (1.5 μM) were pre-incubated with Si NWs (40.5–324 μg/mL) for 2 h before being applied to PC12 cells for 24 h. Data are presented as mean ± standard error of mean (*n* = 6). Statistical significance was assessed using one-way ANOVA followed by Tukey’s post hoc test, with **** *p* < 0.0001 and * *p* < 0.05 denoting significant differences.

The optimal concentration of A*β*_1–42_ oligomers for the experiment was determined. PC12 cells were incubated with varying concentrations (0.75, 1.5, and 3 μM) of A*β*_1–42_ oligomers for 48 h, followed by MTT assay. Compared to the vehicle control (normal cell growth), cell viability decreased to 94.0%, 38.5%, and 34.4%, respectively (Figure 4a). The ideal condition aimed for at least 50% cell death in PC12 cells. Therefore, 1.5 μM A*β*_1–42_ oligomers were chosen as the optimal concentration for further experiments. To assess the biocompatibility of Si NWs, PC12 cells were incubated with different concentrations (20.25–324 μg/mL) for 24 h. MTT assay revealed nearly 100% cell viability across all Si NW groups (Figure 4b), indicating good biocompatibility and minimal toxicity. Finally, PC12 cells were pre-treated with Si NWs for 2 h, followed by addition of 1.5 μM A*β*_1–42_ oligomers. This protocol enabled investigating the potential protective effect of Si NWs against A*β*_1–42_-induced cell damage. Figure 4c demonstrates that 1.5 μM A*β*_1–42_ oligomers induced significant cell death (45.6%) compared to the normal growth state. Interestingly, co-incubation with Si NWs (all tested concentrations) resulted in modestly elevated cell viability (64.8–69.0%) compared to the A*β*_1–42_ oligomer group alone. This suggests a potential protective effect of Si NWs against A*β*_1–42_-induced cytotoxicity. Further supporting this notion, Figure 4d shows a more pronounced protective effect when Si NWs were pre-incubated with A*β*_1–42_ oligomers for 48 h before the addition to PC12 cells. While the A*β*_1–42_ oligomer group exhibited only 36.5% cell viability after 24 h, pre-treatment with Si NWs significantly increased viability to 80.2%. These results imply that Si NWs can not only mitigate the toxicity of newly formed A*β*_1–42_ oligomers but also potentially reduce the harm caused by existing oligomers. However, the observed protective effect appears to be more pronounced against newly formed oligomers (Figure 4d) compared to pre-existing ones (Figure 4c). This suggests that Si NWs may be more effective in preventing A*β*_1–42_ oligomerization than modifying the toxicity of already formed oligomers.

**Figure 5:**
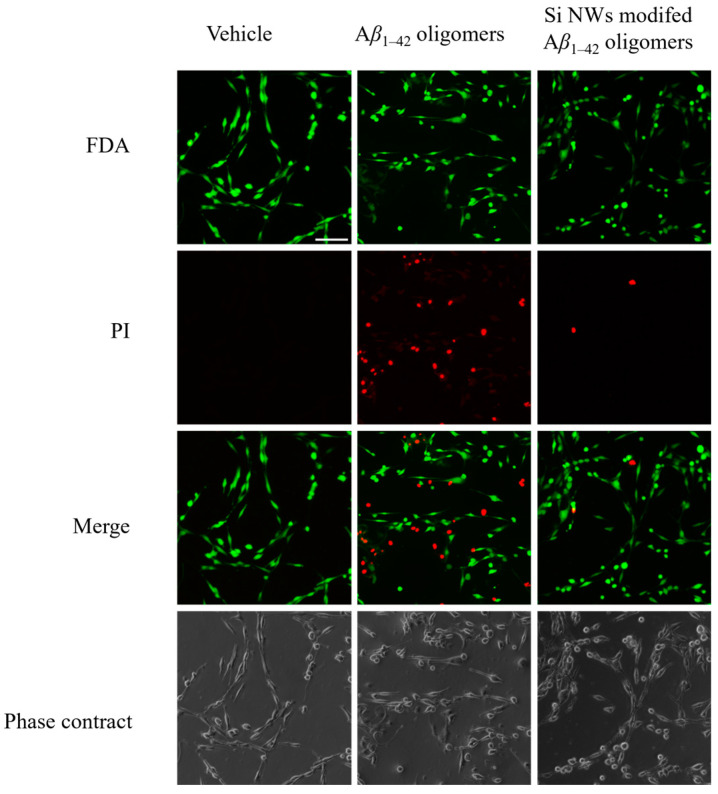
Addition of Si NWs (324 μg/mL) attenuated cell death induced by A*β*_1–42_ oligomers (1.5 μM), as confirmed by FDA/PI double staining. Scale bar: 20 μm. Green fluorescence indicates the live cells while the red fluorescence represents the dead cells.

To further confirm the MTT assay results (Figure 4), we employed live/dead cell discrimination using FDA/PI double staining (Figure 5). As expected, the PBS control group exhibited vibrant green fluorescence (indicating live cells labeled by FDA) without red fluorescence (dead cells labeled by PI). Conversely, A*β*_1–42_ oligomer treatment significantly weakened green fluorescence while intensifying red fluorescence, signifying cell death induction. This finding strengthens the evidence for A*β*_1–42_ oligomer-induced cytotoxicity observed in the MTT assay. Double staining with FDA and PI dyes provided further evidence for the cytotoxicity of A*β*_1–42_ oligomers and the neuroprotective potential of Si NWs. As expected, the control group treated only with PBS displayed robust green fluorescence due to FDA labeling of healthy cells. No red fluorescence, which indicates dead cells stained by PI, was observed. In contrast, A*β*_1–42_ oligomer treatment significantly weakened green fluorescence and intensified red fluorescence, revealing a strong apoptotic response. This observation visually corroborates the A*β*_1–42_ oligomer-induced cytotoxicity highlighted by the MTT assay (Figure 4). Importantly, co-incubation with Si NWs at 324 μg/mL reversed this trend. Green fluorescence intensity increased notably, suggesting preservation of viable cells, while red fluorescence diminished, indicating a reduction in apoptosis. This visual evidence aligns with the previously observed protective effect of Si NWs in the MTT assay, further strengthening the conclusion that Si NWs can effectively attenuate A*β*_1–42_ oligomer-induced cytotoxicity.

### 2.4. Discussion

Exposure to air leads to the formation of a stable SiO_2_ oxide layer on the surface of Si NWs under dry conditions. However, this layer readily dissolves in aqueous solutions, particularly under high ionic strength [41]. Furthermore, at the physiological pH of 7.4, Si NWs with an isoelectric point (pI) around 5 [42] acquire a negative charge. This negative charge generates electrostatic interactions with cations within the A*β*_1–42_ polypeptide chain. Additionally, the A*β*_1–42_ monomer itself also carries a net negative charge at pH 7.4 due to its pI of 5.5. Consequently, electrostatic forces attract A*β*_1–42_ monomers to the negatively charged periphery of Si NWs. This interaction not only promotes A*β*_1–42_ oligomerization but also facilitates fibril nucleation on the Si NW surface, potentially interfering with the amyloid fibrillization process.

**Figure 6:**
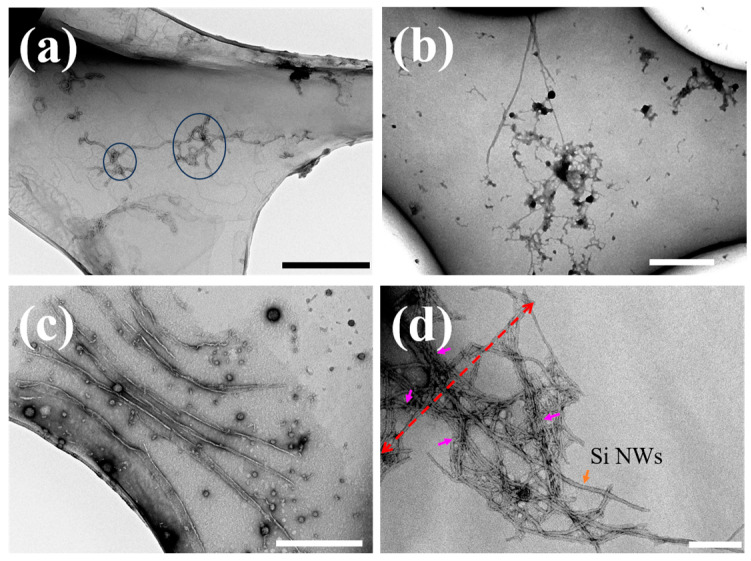
(**a**) TEM image of A*β*_1–42_ monomer (10 μM) incubated with PBS (Vehicle) for 24 h under fibrotic conditions (scale bar 500 nm). TEM images of A*β*_1–42_ monomer (10 μM) incubated with PBS (Vehicle) and Si NWs (324 μg/mL) for different times under fibrotic conditions: (**b**) 0 h (scale bar 500 nm); (**c**) 1 h (scale bar 500 nm); (**d**) 24 h (scale bar 200 nm). The red dashed arrow indicates the measurement position of the maximum width of the aggregate. The purple arrow indicates the position for randomly selected measurements of aggregate width with Si NWs as the core.

While AFM demonstrated significant inhibition of A*β*_1–42_ fibrillization by Si NWs, their visualization within the aggregates remained elusive. During the preparation of the AFM sample, we agitated the liquid samples firstly by using a vortex mixer. Subsequently, we selected 2 μL of clear solution from the center of the mixer to drop onto the mica sheet. To avoid the degradation of the AFM observation quality due to the loose salt accumulation, we rinsed the mica surface three times with deionized water. This process, however, led to the loss of Si NWs, making the visualization of Si NWs a challenge. To gain deeper insights into their interaction and remodeling effects, we employed TEM to image the association of Si NWs with A*β*_1–42_ monomers, oligomers, and amyloid fibrils (Figure 6). Figure 6a showcases the morphology of amyloid fibrils formed from A*β*_1–42_ monomers incubated under fibrillating conditions at 37 °C for 24 h. In the absence of Si NWs, the image reveals a dense network of interwoven long fibrils, characteristic of mature A*β*_1–42_ aggregates [5]. This observation serves as a baseline for comparison with the Si NW-treated samples. To elucidate the temporal interactions between Si NWs and A*β*_1–42_, A*β*_1–42_ monomers (10 μM) were incubated with PBS (control) or 324 μg/mL Si NWs under fibrillating conditions for 0, 1, and 24 h before TEM imaging (Figure 6b–d). Both monomers and oligomers were observed in close proximity to Si NWs throughout the incubation period (Figure 6b,c), suggesting peptide attraction to NWs. This localized concentration increase likely facilitates rapid nucleation and elongation of A*β*_1–42_ fibrils. Interestingly, compared to control A*β*_1–42_ fibrils presented in Figure 6a, short fibrils wrapping around Si NWs were observed after 24 h (Figure 6d). The absence of junctions similar to those shown in Figure 6a (blue circles) indicates that Si NWs influence fibril morphology by promoting shorter, less entangled structures. To further characterize the Si NW-A*β*_1–42_ complexes observed in Figure 6d, we performed local size measurements. Considering the entire complex as a single entity yielded a width of approximately 837 nm (red dashed arrow), inconsistent with the particle size distribution observed in AFM images. However, measuring the width with Si NWs as the core (small pink arrows) at four random locations within Figure 6d produced consistent results within the distribution range, with an average value of 60.3 nm. This diameter closely aligns with the distribution of Si NWs characterized in Figure 1c, supporting the interpretation that A*β*_1–42_ wraps around the surface of Si NWs rather than forming large, mixed aggregates. This distinction suggests that the AFM images likely capture aggregates of unbound peptides freely dispersed in the solution. Therefore, decreasing Si NW concentrations would result in a greater abundance of unbound peptides, leading to the formation of larger protein patches observed in AFM.

**Figure 7:**
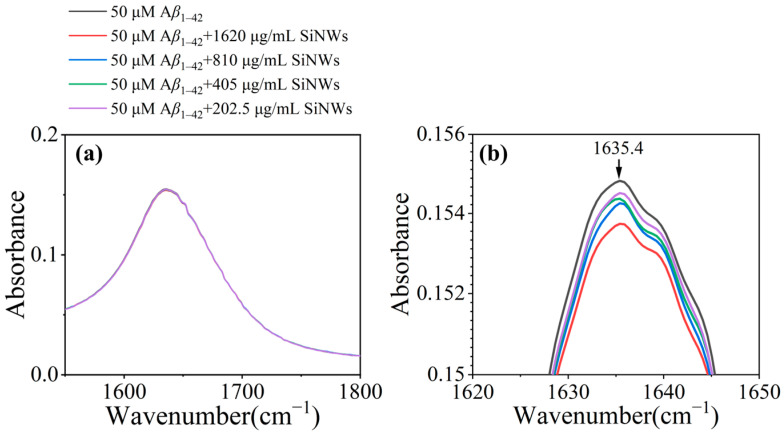
(**a**) ATR-FTIR spectra of A*β*_1–42_ original fibrils at a concentration of 50 μM co-cultured with Si NWs at different concentrations for 24 h. (**b**) Detailed FTIR spectra in the spectral range of 1620~1650 cm^−1^.

Figure 7 presents the FTIR spectra of the complexes formed by A*β*_1–42_ fibrils at a concentration of 50 μM in the absence or presence of different concentrations of Si NWs. The presence of a strong absorption peak at 1635.4 cm^−1^ in the sample with only A*β*_1–42_ aggregates (Figure 7a) indicates the presence of a major cross-*β*-sheet structure in the amyloid-like protein aggregates. The literature reported that the absorption spectra of amide I and II bands depend on changes in the *β*-amyloid secondary structure [43]. The spectral features of proteins with natural *β*-sheet structure clusters are in the range of 1630~1643 cm^−1^ [44]. The details of the spectra at the position of 1635.4 cm^−1^ are enlarged, as shown in Figure 7b. With the addition of Si NWs, a noticeable decrease in the absorption peak at 1635.4 cm^−1^ is observed as compared to the absorption peak of A*β*_1–42_ alone. This demonstrates that the original *β*-sheet structure of the amyloid-like protein is disrupted in the presence of Si NWs. Figure 7b also shows the intervention of different concentrations of Si NWs in the aggregation of *β*-amyloid proteins. As the concentration of Si NWs decreases, the absorption peak gradually increases, proving that the ability of Si NWs to intervene in the formation of *β*-sheet structures of amyloid-like proteins weakens as the concentration of Si NWs decreases.

## 3. Experimental Methods

### 3.1. Synthesis and Transfer of Si NWs

Prior to Si NW growth via VLS mechanism, 2 nm Au films were deposited onto Si wafers using electron beam evaporation. It should be noted that Au exists only at the tip of NWs after NW growth. The Si NWs used here are pure Si due to the incorporation of Au into Si NWs with an ultralow content of 0.001 at. % [45]. To promote optimal growth conditions, the wafers underwent an initial annealing step within a PECVD system. This involved passing 100 sccm of H_2_ at 550 °C and 130 Pa for 30 min. Subsequently, the pressure was increased to 150 Pa, and SiH_4_ (10 sccm) and H_2_ (200 sccm) were introduced while applying 10 W of RF power for 10 min, facilitating the VLS growth of Si nanowires. Note here that Si NWs in this work have structures of crystalline Si cores covered by amorphous Si shells [46]. Following NW synthesis, ultrasonic oscillation in pure water, PBS solution, or absolute ethanol as selected carriers, efficiently detached the Si NWs and enabled their transfer to the suspension medium. The transferred mass of Si NWs was quantified by gravimetric analysis, subtracting the sample mass before and after sonication. Finally, the suspension volume was adjusted to achieve the desired concentration, followed by subsequent dilutions to generate stock solutions of varying Si NW concentrations.

### 3.2. Preparation of Aβ_1–42_ Fibrils

The method for preparing A*β*_1–42_ fibrils is as follows [47]. A*β*_1–42_ powder (GL Biochem, Shanghai, China) was dissolved in hexafluoroisopropyl alcohol (HFIP, Sigma, St. Louis, MO, USA) to form a monomer solution. Subsequently, 100 µL of the A*β*_1–42_ monomer solution was diluted with 60 µL of 20 µM sodium hydroxide, triggering fibrillization. HFIP was then volatilized using a N₂ stream to obtain a final A*β*_1–42_ monomer concentration of 1 mM. For Si NW treatment, 2 µL of the 10 µM A*β*_1–42_ monomer solution was incubated with 197 µL of PBS (control) or 197 µL containing 10 µL of Si NWs (40.5–324 µg/mL) for 24 h at 37 °C. This resulted in a final A*β*_1–42_ concentration of 50 µM in all samples. It should be emphasized that the freeze-dried powder used in the sample was pre-treated, and there was almost no pre-aggregate before this experiment, and the freeze-dried powder added into the experiment was already in monomer form. In the experimental step, lyophilized powder dissolved in HFIP was already redissolved, and this action of redissolution is also to ensure that there is no pre-polymerization before in the aqueous environment. During the process of lyophilized powder dissolving in HFIP, when it was almost completely dissolved, it was frozen at −20 °C, so there was no pre-polymerization [48]. The study also found that the behavior of N_2_ blowing can completely remove a small amount of HFIP [49] in the water phase, and there is a water phase environment in the process of sample preparation, which may produce seeds. However, all the samples required for the experiment were prepared and used at the time, but the time interval was very short, which has little impact on the experimental results.

### 3.3. Preparation of Aβ_1–42_ Oligomers

A*β*_1–42_ powder was dissolved in HFIP to prepare a monomer solution. Subsequently, 100 µL of the A*β*_1–42_ monomer solution was diluted with 900 µL of Milli-Q water, triggering oligomer formation. HFIP was then volatilized using a gentle N₂ stream to yield a final A*β*_1–42_ monomer concentration of 50 µM. These A*β*_1–42_ oligomer solutions were further incubated with varying concentrations of Si NWs (40.5–324 µg/mL) at a final A*β*_1–42_ concentration of 10 µM. All samples were maintained at room temperature with continuous gentle vibration to promote and maintain the predominantly oligomeric state of A*β*_1–42_.

### 3.4. ThT Fluorescence

To assess A*β*_1–42_ fibril formation, 200 μL of each incubation sample was mixed with 0.5 μL of 10 mM ThT (Sigma Aldrich, Shanghai, China) in a Costar 96-well plate with black/clear bottoms. The plate was incubated at 37 °C with shaking at 120 rpm. ThT fluorescence intensity was measured at each time point using a Thermo Scientific (Madison, WI, USA) Fluoroskan Ascent FL fluorescence plate reader set to 440 nm excitation and 485 nm emission. Data acquisition and analysis were performed using Ascent Software for Fluoroskan Ascent software ver 2.6.

### 3.5. TEM Imaging

To evaluate interactions between Si NWs and A*β*_1–42_, 10 μL of 324 μg/mL Si NW solution was mixed with 2 μL of 10 μM A*β*_1–42_ monomer solution and incubated at 37 °C with shaking. This 209 μL mixture, representing fresh monomer + Si NWs, was then diluted with 197 μL water to create samples for three time points:

- 0 h A*β*_1–42_: Undiluted mixed solution representing initial conditions.
- 1 h A*β*_1–42_: A 1:1 (*v*/*v*) dilution representing 1 h incubation (oligomers).
- 24 h A*β*_1–42_: A 1:2 (*v*/*v*) dilution representing 24 h incubation (fibrils).

Lower portions of these samples were pipetted onto 200-mesh copper grids with carbon film (Mesopicroscope instrument, Zhongjing Keyi Technology, Beijing, China) pre-treated with 30 s of glow discharge. Samples were adsorbed for 60 s, excess liquid was removed with filter paper, and the grid was cleaned with 10 μL of deionized water. Negative staining was achieved with 5 μL of 1% uranyl acetate for 30 s, followed by excess stain removal and air drying. Characterization was performed using a HITACHI HT7800 transmission electron microscope (AMT, Laurens, SC, USA) at 80.0 kV.

### 3.6. AFM Imaging

To analyze interactions between Si NWs and A*β*_1–42_, 10 μL of Si NW solution (40.5–324 μg/mL) was mixed with 2 μL of 10 μM A*β*_1–42_ monomer solution. This 202 μL mixture was diluted with 197 μL PBS solution and incubated at 37 °C with shaking at 120 rpm for 24 h. Following incubation and final shaking, a 2 μL droplet was collected from the central liquid portion and deposited onto a clean mica sheet after dissociation. This sample was incubated for 30 min at room temperature. To remove unbound species and excess salt, the mica flake was washed three times with Milli-Q water. Gentle nitrogen gas flow was then used to remove residual water.

### 3.7. PC12 Cell Culture

PC12 cells were obtained from the Shanghai Institute of Cell Biology (Chinese Academy of Sciences, Shanghai, China). PC12 cells were routinely cultured in high-glucose modified Eagle’s medium (DMEM) supplemented with 10% fetal bovine serum (FBS) and penicillin (100 U/mL)/streptomycin (100 μg/mL). Cultures were maintained at 37 °C in a humidified atmosphere with 5% carbon dioxide, and the medium was replaced every two days. Twenty-four hours prior to experimentation, the complete growth medium was replaced with DMEM containing only 1% FBS. This serum starvation step is commonly employed to enhance cellular responsiveness to various stimuli, including growth factors and neurotransmitters.

### 3.8. Measurement of Cell Viability

Cell cytotoxicity was evaluated using the 3-(4,5-dimethylthiazol-2-yl)-2,5-diphenyltetrazolium bromide (MTT) assay. Five microliters of Aβ_1–42_ aggregates, with or without various Si NW concentrations, were added to 96-well plates containing 100 microliters of fresh culture medium. Following co-incubation with cells for 24 h at 37 °C, 10 microliters of MTT solution were added. After a further 4 h of incubation, the medium was discarded and 100 microliters of a dissolving solution (0.01 M HCl in 10% SDS) were added to solubilize the formazan crystals produced by metabolically active cells. After 16 h, the absorbance was measured at 570 nm with a reference wavelength of 655 nm using a microplate reader.

### 3.9. FDA/PI Double Staining Method

Cell viability after exposure to A*β*_1–42_ aggregates with or without Si NWs was determined using the fluorescein diacetate (FDA)/propidium iodide (PI) double staining technique. Briefly, 100 μL of A*β*_1–42_ aggregates was added to cultured cells. Following a 6 h co-incubation at 37 °C, the medium was discarded, and cells were stained with a pre-mixed FDA/PI solution (5 μg/mL PI, 10 μg/mL FDA) for 15 min in the dark. Subsequently, stained cells were observed and imaged under UV and phase-contrast microscopy. Five random fields were captured per well. Cell viability was calculated as the percentage of FDA-positive cells relative to the total cell number (FDA-positive + PI-positive). This calculation reflects the proportion of metabolically active (viable) cells in the population.

### 3.10. ATR-FTIR Spectroscopy

The A*β*_1–42_ monomer (50 μM) and Si NWs with concentrations of 1620~202.5 μg/mL were co-incubated in a fibrillization system (200 μL) at 37 °C with a constant shaking, using PBS solution (pH = 7.3) as the solvent. After 24 h, the samples were measured by FTIR with the sample dropped onto the surface of the ATR crystal. ATR-FTIR spectra were collected using a Nicolet™ iS™ 10 FTIR spectrometer (Thermo Fisher Scientific, Madison, WI, USA) equipped with a Smart iTR™ attenuated total reflectance accessory. The spectra for each sample were obtained based on the background spectra collected from air.

## 4. Conclusions

In summary, the ThT fluorescence intensities of Si NWs co-incubated with Aβ1–42 monomers decreased with increasing Si NWs concentrations. The concentration dependence test results showed that even if the concentration of Si NWs is 40.5 μg/mL, a 78% inhibition result can still be obtained. In vitro experiments showed that with the addition of 324 μg/mL Si NWs to the environment of existing A*β*_1–42_ oligomers, the cell survival rate was 65.4%, while with the addition of 324 μg/mL Si NWs and A*β*_1–42_ oligomers, the cell survival rate increased to 80.2%. Si NWs mitigated the toxicity of A*β*_1–42_ oligomers. However, regarding their multi-interactions with A*β*_1–42_ monomers, we suspect that they include hydrogen bonding, electrostatics, and hydrophobicity.

## Data Availability

Data are contained within the article.
